# Infantile Asthma

**Published:** 1915-09

**Authors:** John C. Warbrick

**Affiliations:** Chicago


### BUFFALO MEDICAL JOURNAL

Yearly Volume 71 SEPTEMBER, 1915 No. 2

### ORIGINAL ARTICLES

The right is reserved to decline papers not dealing with practical medical and surgical subjects, and such as might offend or fail to interest readers. Contributors are solely responsible for opinions, methods of expression and revision of proof.

### Infantile Asthma.

By JOHN C. WARBRICK, M. D., Chicago.

One of the principal causes producing infantile asthma would seem to be the common condition mostly found in the young, and well-known everywhere by the common name of adenoids. These are kind of soft masses of gelatinous-like or lymphoid tissue found in the post nasal space at the back of the posterior nares where they form an obstruction to proper breathing; in some cases, of course, a good deal more than in others, thus tending to produce an asthmatic state, or that of some trouble in breathing.

Anything tending to cause obstruction in the nasal passages of a child of any age, of course, is likely to produce more or less of an asthmatic condition, and often it does not take very much of an obstruction on account of the nasal passages being very narrow, small, and readily capable of getting blocked up in children, and almost the same of the rest of the respiratory tract; more so the bronchial tubes, which can be easily choked up with mucous.

When children are young it often does not take much to get the mucous membrane of the pharynx, larynx and bronchial tubes out of order, more so if they are stuffed continually with all kinds of food, much beyond what is required for their system. Under such conditions there may be a tendency to excessive secretion of mucous in the respiratory tract or some part of it, as in the nose or throat, thus the mucous membrane gets into a catarrhal state, or the child may get a cold or a cough.

It is not very easy then for a child to throw off the mucous accumulated from time to time, and so an asthmatic condition may be brought about.

If the above state is associated at any time with adenoids it, of course, only tends to make it worse and more serious.

By removing the adenoids the existing trouble can, in most cases, be relieved. Another thing likely to obstruct the nasal passages, and cause more or less trouble in breathing, is the presence of a nasal polypus or polypi of the mucous variety, the more common of them. These readily acclude the passages and may sooner or later cause trouble, for they tend, in time, to increase in size.

The nostrils may become completely blocked up by the presence of mucous polypi, thus causing the patient to breathe entirely through the mouth. An enlarged turbinal body, either in part or in whole, may also tend to cause an asthmatic condition, and more so if both of them are affected together; while their enlargement may also be associated with the presence of polypi or adenoids.

A deflected septum, either anteriorly or posteriorly, is another form of obstruction which tends to cause asthma in children; the amount of trouble produced depending a good deal upon the amount of displacement and its location. Chronic catarrhal conditions of the nasal passages, or of the post nasal space, are also likely to produce asthma from the mucous membrane getting into a soggy state, thus tending to swell and cause some obstruction.

This may readily extend downwards and cause a more marked degree of it.

Large crusts and scabs may also form in the nose at the same time and help to acclude the passage.

Laryngeal polypi of the mucous form are another cause of asthma in infants or children.

It may be said that catarrhal conditions, which seem to be very common in children from birth up, cause a great deal of trouble along the respiratory tract affecting the mucous membrane, especially that of the bronchial tubes from the fact that they are narrow, the mucous membrane being easily irritated, its feeble resisting power in younger children, and from its lack of strength to throw off any excess of secretion.

The mucous membrane being readily affected by various conditions of the body a catarrhal state can often be brought on so that an asthmatic state may be produced by any accumulated mucous or anything tending to block up the tubes, especially the smaller capillaries, which are more readily affected than those in older persons; thus a suffocative catarrh may be brought on and tend to endanger life by causing an asthmatic spasm.

A clogging up of the system, from a continual overloading of the stomach, or from a state of constipation, may set up catarrhal conditions, which render the susceptible body of young children more liable to chills and colds, thus tending to bring on some bronchial trouble in the feeble resisting organism, such as capillary bronchitis, which may cause a great deal of difficulty in breathing, and thus, in time, endanger life.

A slight catarrhal affection may persist for some time in children and not be detected.

It may get more severe and manifest itself in many ways, especially in the bronchial form, while it tends to lower the resisting powers of the child, and the same of nasal catarrh, in time.

Another cause of infantile asthma that may bring on a sudden spasm, and one that should not be overlooked, is an overloaded stomach, or the presence of indigestible food, such as unchewed meat, producing more or less distension.

From almost any continual irritation the child may get a sudden spasm, bringing on choking and suffocation at once, causing a high pulse rate anywhere from 120 to 140, making it blue in the face, with a quick gasping for breath, thus giving some cause for alarm.

Herpes may tend to produce a certain degree of asthma in children, while a temperamental state may bring on a nervous form of it, also a constipated condition which may occur, as it does not seem to take very much sometimes to produce a slight touch of asthma in children, and a mild catarrh or a cold is often sufficient to do this at any time.

An attack of asthma may come on suddenly or not according to what the cause is and the symptoms, as in capillary bronchitis, or catarrh of the suffocative form, or an overloaded stomach. In capillary bronchitis rales will of course be heard over the chest, thus indicating the condition along with breathing.

If the stomach is overloaded from overeating it may cause sickness, and spasms of asthma may come on.

**Treatment**—The cause of the trouble whatever it is should be looked for and removed; such as the presence of adenoids, edema of the glottis, or any nasal obstruction.

The bronchial form should be treated according to symptoms. The bowels should be kept well open as an important point in these cases with children, as any clogging up only tends to aggravate the trouble.

In the capillary form especially is this necessary, and also the catarrhal form tending to suffocation.

Fresh air is important and cold douching may be useful.

In the case of an overloaded stomach causing distress and gasping for breath, the child should be made to vomit at once. This can readily be done by putting the forefinger of right hand down the child's throat and at the same time bending its head downwards. Children should not be stuffed at anytime with all kinds of food such as cakes, pie, meat, or candies. Light nourishing food in the proper amount should be given.

The fumes of nitre should be avoided, also anti-asthmatic cigarettes, and inhalations. The herpetic form should be treated by tonics and cod liver oil. For the spasm morphia sulph 1-30th gr. and atrophine hypod.

Spraying the nose and throat may be useful, also adrenalin chloride used in the nose and silver nitrate applications. In the catarrhal form any congestion of the nasal or pharyngeal mucous membrane should be relieved. This may be done by spraying the nasal passages, the use of adrenalin chloride to contract the blood vessels, and repeated applications of a weak solution of silver nitrate to the front of the nose.

The bowels should be kept well open and a very light diet given or no food at all for a time if required.

---

**Embalming.** Francis, Military Surgeon, Feb. 1915, discusses methods for use on the sea, where long preservation, with perfect safety, is necessary.

| | | |
|---|---|---|
| The formula used is | Formalin (40%) | 125. |
| | Borax | 50. |
| | Water to | 1000. |

An alkali is needed to prevent the bleeding effect of formaldehyde. Borax was found to be the only one that would not cause deterioration of the formaldehyde and is itself a preservative. This solution has been found to keep for three years. Single-artery injection is inefficient—as often noted in everyday experience with "embalmed" bodies. About 15% of the body-weight should be injected, remembering that a pound is 453.59 grams. 2% should be injected into each femoral toward the toes, 1% into each brachial, toward the fingers, 2% into one common carotid toward the head, 7% into the same toward the heart. It is not necessary to drain the veins nor to inject the body cavities, although these should be drenched with the fluid and cotton or saw-dust used for filling in case of necropsy. The anus, mouth and nostrils should be plugged with cotton soaked in the embalming fluid. The entire surface of the body should be washed with it. To preserve for a long time against dessication, rub vaseline liberally over the surface and wind with bandages. Injection is facilitated by using a 3-gallon bottle, with rubber stopper admitting two connections, one for a glass canula to be inserted into the arteries, the other for a pump—a bicycle foot-pump working well. Too much fluid causes a sort of dropsy which, however, disappears on long preservation. This method has been proved efficient after two months of exposure to a temperature of 98 degrees, after embalming.

